# Adaptation Study of the Genital Appearance Satisfaction Scale into Turkish

**DOI:** 10.1192/j.eurpsy.2025.2424

**Published:** 2025-08-26

**Authors:** D. Ocaklik Altunkiliç, H. Bilgin

**Affiliations:** 1 Mental Health and Psychiatric Nursing, Bakırköy Mazhar Osman Mental Health and Neurological Diseases Education and Research Hospital; 2 Mental Health and Psychiatric Nursing, Istanbul University-Cerrahpasa , Istanbul, Turks and Caicos Islands

## Abstract

**Introduction:**

As a result of dissatisfaction with genital appearance, even the most intimate genital organs have become open to change, leading to the emergence of female genital cosmetic surgery (FGCS). Although there is known to be a significant increase in the number of women seeking these procedures, the psychosocial aspects and motivations of women undergoing these procedures are not fully understood. It is believed that satisfaction with genital appearance is related to body satisfaction, self-esteem and the importance placed on appearance. Many women are known to have negative perceptions regarding their genital organs.

**Objectives:**

Given the lack of research on the subject in our country and the need to better understand women’s perceptions of their own genital appearance, the primary aim of this study is to examine the validity and reliability of the Genital Appearance Satisfaction Scale in Turkish women. In this context, it is planned to conduct the Turkish adaptation of the “Genital Appearance Satisfaction Scale” in order to identify the factors contributing to the development of genital appearance dissatisfaction in women and to determine the current state of the issue.

**Methods:**

The research is a descriptive, methodological and cross-sectional study. The population consists of women aged 18-65 and data were collected through a web-based survey. First, the scale was translated from English to Turkish and after evaluation by experts, it was back-translated. The translations were compared and presented for expert opinion. The scale was created by applying content validity. To test the clarity of the items, a pilot study was conducted and the final version of the scale was formed. The data obtained from the research were analyzed using SPSS 24 with descriptive statistics, analyses required for psychometric standardization (item correlations, factor analysis, internal consistency coefficient, etc.), comparison and correlation statistics. The construct validity of the scale was tested using confirmatory factor analysis with the AMOS program. The test-retest measurements of the scale were tested with a paired t-test and correlation analysis.

**Results:**

A reliability analysis was applied to the scale, and the Cronbach’s Alpha coefficient was found to be 0.83. The Cronbach’s Alpha values for the subscales were calculated as follows: 0.88 for the “Genital Appearance” subscale, 0.85 for the “Impact of Genital Satisfaction on Daily Life” subscale, and 0.85 for the “Impact of Genital Satisfaction on Sexual Life” subscale.

**Conclusions:**

The fit indices calculated via confirmatory factor analysis showed that the scale is acceptably compatible with the previously identified factor structure. When the standardized coefficients were examined, it was found that the factor loadings were high, standard error values were low and t-values were significant. These results confirmed the construct validity of the previously identified factor structure.

**Disclosure of Interest:**

None Declared

